# CRISPR/Cas9 mediated editing of the Quorn fungus *Fusarium venenatum* A3/5 by transient expression of Cas9 and sgRNAs targeting endogenous marker gene *PKS12*

**DOI:** 10.1186/s40694-021-00121-8

**Published:** 2021-11-17

**Authors:** Fiona M. Wilson, Richard J. Harrison

**Affiliations:** 1 NIAB EMR, New Road, East Malling, West Malling, Kent, ME19 6BJ UK; 2 NIAB, 93 Lawrence Weaver Road, Cambridge, CB3 0LE UK

**Keywords:** AMA1, CRISPR/Cas9, Codon optimisation, *Fusarium venenatum*, PolII promoter, *5SrRNA*

## Abstract

**Background:**

Gene editing using CRISPR/Cas9 is a widely used tool for precise gene modification, modulating gene expression and introducing novel proteins, and its use has been reported in various filamentous fungi including the genus *Fusarium*. The aim of this study was to optimise gene editing efficiency using AMA1 replicator vectors for transient expression of CRISPR constituents in *Fusarium venenatum* (A3/5), used commercially in the production of mycoprotein (Quorn™).

**Results:**

We present evidence of CRISPR/Cas9 mediated gene editing in *Fusarium venenatum*, by targeting the endogenous visible marker gene *PKS12*, which encodes a polyketide synthase responsible for the synthesis of the pigment aurofusarin. Constructs for expression of single guide RNAs (sgRNAs) were cloned into an AMA1 replicator vector incorporating a construct for constitutive expression of *cas9* codon-optimised for *Aspergillus niger* or *F. venenatum*. Vectors were maintained under selection for transient expression of sgRNAs and *cas9* in transformed protoplasts. 100% gene editing efficiency of protoplast-derived isolates was obtained using *A. niger cas9* when sgRNA transcription was regulated by the *F. venenatum 5SrRNA* promoter. In comparison, expression of sgRNAs using a *PgdpA*-ribozyme construct was much less effective, generating mutant phenotypes in 0–40% of isolates. Viable isolates were not obtained from protoplasts transformed with an AMA1 vector expressing *cas9* codon-optimised for *F. venenatum*.

**Conclusions:**

Using an AMA1 replicator vector for transient expression of *A. niger cas9* and sgRNAs transcribed from the native *5SrRNA* promoter, we demonstrate efficient gene editing of an endogenous marker gene in *F. venenatum*, resulting in knockout of gene function and a visible mutant phenotype in 100% of isolates. This establishes a platform for further development of CRISPR/Cas technology in *F. venenatum* for use as a research tool, for understanding the controls of secondary metabolism and hyphal development and validating prototypes of strains produced using traditional methods for strain improvement.

**Supplementary Information:**

The online version contains supplementary material available at 10.1186/s40694-021-00121-8.

## Background

Quorn™ myco-protein is a high fibre, high quality protein with a significantly lower environmental impact compared to that of beef, produced by fermentation of *Fusarium venenatum* (ATCC 20334, A3/5) utilising wheat-derived glucose in a continuous fermentation process. The organism was selected for its efficient biomass conversion, nutritional value, sparsely branched mycelium which enables processing into a meat-like texture and absence of undesirable secondary metabolites under optimal growth conditions [1–3]. *F. venenatum* has no known sexual cycle and therefore strain improvement is only currently possible thorough clonal mutagenesis. There are multiple opportunities for strain improvement, including use of alternative carbon sources, which may require strain development for optimal growth and mycoprotein quality. These advances could be facilitated by biotechnological innovations including development of gene editing mediated by CRISPR/Cas9, for use currently as a research tool, and possibly for use in future as a method for strain improvement.

The aim of the current study was to optimise CRISPR/Cas9 mediated gene editing in *F. venenatum* and establish a platform for its use in understanding genetic controls of metabolic pathways and hyphal development. CRISPR/Cas9 is a well-established technology, successfully used in a wide range of organisms for precise gene editing, gene insertion and modulation, facilitating genomic functional analyses and production of novel compounds. The Cas9 enzyme, guided by a sequence-specific RNA complex (sgRNA), generates a double stranded break at the target site in the genome, which can be repaired by either the non-homologous end joining (NHEJ) or microhomology –mediated end joining mechanisms (which are error prone), or by homology-directed repair (HR) in the presence of a repair template [4, 5].

CRISPR/Cas9 mediated gene editing in filamentous fungi was first reported in *Aspergillus* [6, 7], *Neurospora crassa* [8] and *Trichoderma reesei* [9] and subsequent reports include use of CRISPR/Cas9 in *Penicillium chrysogenum* [10], *Ustilago maydis* [11] and *Magnaporthe oryzae* [12]. Its application in *Fusarium* was reported initially in *F. graminearum* [13] and *F. oxysporum* [14] and subsequently in *F. proliferatum* [15] and *F. fujikuroi* [16].

Parameters for optimising the methodology in fungi have included use of various delivery systems for CRISPR constituents, evaluation of promoters driving sgRNA expression and codon optimisation of *cas9* and its fusion to appropriate protein nuclear localization signals (NLS).

Strategies used for delivery of CRISPR constituents in filamentous fungi including *Fusarium* include generation of stable transgenic lines for expression of *cas9* and sgRNAs [6, 12, 13], introduction of in vitro expressed sgRNAs [9, 16, 17], introduction of Cas9/sgRNA ribonucleic protein (RNP) complexes [10, 12, 14, 15, 17, 18], and transformation with expression vectors for transient production of Cas9 and sgRNAs in vivo [8, 16]. Additionally, sgRNAs produced in vitro have been used in *Aspergillus* [19] and AMA1 replicator vectors for transient expression of sgRNAs and *cas9 *in vivo have also been used successfully [7, 10, 11], but their use has not been reported for the genus *Fusarium*.

PolIII promoters are commonly used to drive sgRNA expression in fungi including *Fusarium*, such as the yeast SNR52 promoter [6, 8] and endogenous promoters for *U6* [9–11, 16, 18], tRNA [10], *U3* [20] and *5SrRNA* [16, 19], and in *Aspergillus* and *Fusarium* sgRNAs were embedded in a dual ribozyme driven by a PolII promoter [7, 13].

Successful editing in fungi has been achieved using Cas9 proteins optimised for other organisms, such as human-optimized Cas9 (hSpCas9) [6, 8, 12, 15, 18] and *A. niger* optimised Cas9 for gene editing in *F. oxysporum* [14], or Cas9 optimised for the target organism including *Fusarium* [7, 9, 13, 16].

We report on use of the AMA1 vector system for transient expression of *cas9* and sgRNAs via protoplast transformation, which has not previously been reported for *Fusarium,* and evaluate gene editing efficiency of sgRNAs transcribed using both an endogenous *5SrRNA* promoter (*PFv5SRNA*) and the PolII *gdpA* promoter (*PgdpA*) dual ribozyme system. We additionally compare gene editing efficiency of a *F. venenatum* codon optimised Cas9 (*Fv* Cas9) with Cas9 optimised for *A. niger* (*A. niger* Cas9), which has previously been used for gene editing of *Fusarium* [12].

## Results

### CRISPR vectors

**Fig. 1:**
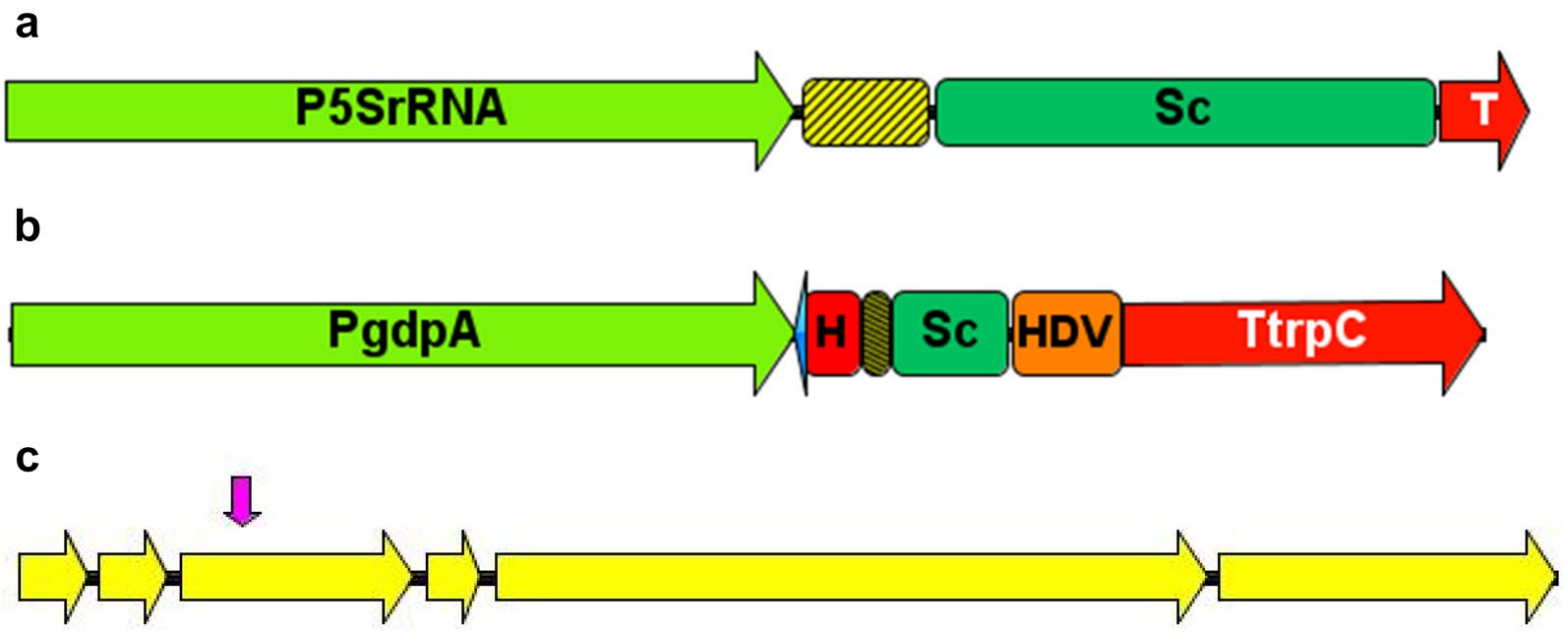
Schematic maps of single guide RNA (sgRNA) expression constructs and the *Fusarium venenatum PKS12 *gene indicating the target site used for CRISPR/Cas9 editing. **A** *Fv5SrRNA *promoter-sgRNA construct, showing positions of *PFv5SrRNA*, 20 base target site sequence (yellow and black striped box), scaffold sequence (Sc) and terminator (T). **B** *PgdpA *dual ribozyme-sgRNA construct showing positions of *PgdpA*, inverted repeat (blue arrow) of first six bases in target sequence (yellow and black box), HH and HDV ribozyme sequences and *trpC* terminator (TtrpC). **C ***F. venenatum* *PKS12 *gene showing exons (yellow arrows) and the location of the CRISPR target sequence (pink arrow) in exon 3, 3-14 (‘14’) antisense strand, sequence position 3’–5’ = 238-257

sgRNAs targeting the endogenous *PKS12* marker gene were assembled with *PFv5SRNA* or *PgdpA* (Fig. 1), for transcription from AMA1 vectors expressing either *Fvcas9* (pFCFvCas9::5S-PK3/14) or *A. niger cas9* (pFC332::5S-PK3/14 and pFC332::PolII-PK3/14).

### Transformants obtained using CRISPR vectors, phenotypes and sequence analysis of isogenic isolates

A replicated experiment was performed to obtain transformed protoplast derived colonies, for a comparison of editing efficiency of *Fvcas9* with *A. niger cas9*, and to compare efficacy of sgRNA promoters *PFv5SrRNA* and *PgdpA*. For each experiment three replicate transformations were performed with each vector and after 10 days the number of mycelial colonies observed on transformation plates was recorded (Table 1). A total of only 2 colonies developed from protoplasts transformed with empty vector pFCFvCas9 expressing *Fvcas9*, compared to 35 from protoplasts transformed with empty vector pFC332 expressing *A. niger cas9* and only one colony developed from protoplasts transformed with pFCFvCas9::PolII-PK3/14. Using pFC332 vectors, fewer colonies developed when sgRNAs were transcribed from the *Fv5SrRNA* promoter compared to *PgdpA*: a total of 12 transformants were obtained using pFC332::5S-PK3/14 compared to a total of 55 following transformation with pFC332::PolII-PK3/14.

**Table 1:** Numbers of colonies regenerating from protoplasts of *F. venenatum* transformed with AMA1 ‘CRISPR’ vectors

Vector	CRISPR constituents	Experiment 1				Experiment 2			
		A	B	C	Total	A	B	C	Total
pFCFvCas9	*Fv* Cas9	0	1	0	**1**	0	1	1	**2**
pFC332	*A.niger* Cas9	11	0	1	**12**	14	6	3	**23**
pFCFvCas9::PolII-PK3/14	*Fv* Cas9 *PFv5SrRNA*-sgRNA	0	0	0	**0**	1	0	0	**1**
pFC332::5S-PK3/14	*A.niger* Cas9 *PFv5SrRNA*-sgRNA	0	1	0	**1**	2	6	3	**11**
pFC332::PolII-PK3/14	*A.niger* Cas9 *PgdpA*/ribozyme-sgRNA	10	11	4	**25**	16	5	9	**30**

CRISPR constituents were Cas9 codon optimised for *Fusarium venenatum* (*Fv*) or *Aspergillus niger* and sgRNAs targeting the *PKS12* gene were transcribed using PolIII promoter *PFv5SrRNA* or PolII promoter *PgdpA* (*PgdpA*/ribozyme). Numbers given are for colonies counted on selection plates 10 days after transformation of approximately 1 × 10^8^ protoplasts with 5 µg vector DNA. Three replicates (A-C) were performed for each vector transformation and the experiment was repeated (Experiments 1 and 2)﻿.

Viability of colonies taken from transformation experiment 2 was subsequently assessed (Table 2): viable cultures were not recovered from transformants with vectors expressing *Fvcas9* (pFCFvCas9 or pFCFvCas9::5 SPK-3/14), and viability of transformants expressing *A. niger cas9* was less when sgRNAs were transcribed using *PFv5SrRNA* (7 of 12 pFCFvCas9::5SPK-3/14 transformants were viable) compared to transcription from *PgdpA* (all of 27 pFC332::PolII-PK3/14 transformants were viable).

**Table 2:** Viability of *F. venenatum* colonies after transformation with AMA1 CRISPR vectors

Vector	A	B	C
pFCFvCas9	3	0	3 (0)
pFC332	3	2	1 (0)
pFCFvCas9::PolII-PK3/14	1	0	1 (0)
pFC332::5S-PK3/14	12	7	5 (0)
pFC332::PolII-PK3/14	27	25	2 (2)

Viability of colonies from protoplasts transformed with empty CRISPR vectors pFCFvCas9 or pFC332 (expressing *cas9* codon optimised for *F. venenatum* (Fv) or *A. niger*), and vectors with single sgRNA constructs transcribed from the *Fv5SrRNA* promoter (pFC332::5S-PK3/14) or the *PgdpA*-dual ribozyme cassette (pFCFvCas9::PolII-PK3/14 and pFC332::PolII-PK3/14). Column A: numbers of colonies transferred to hygromycin selective medium; Column B: numbers of colonies showing growth after 14 days culture on selective medium; Column C: numbers of colonies not able to grow on selective medium (of these, numbers that subsequently showed growth after transfer to non-selective medium).

**Fig. 2:**
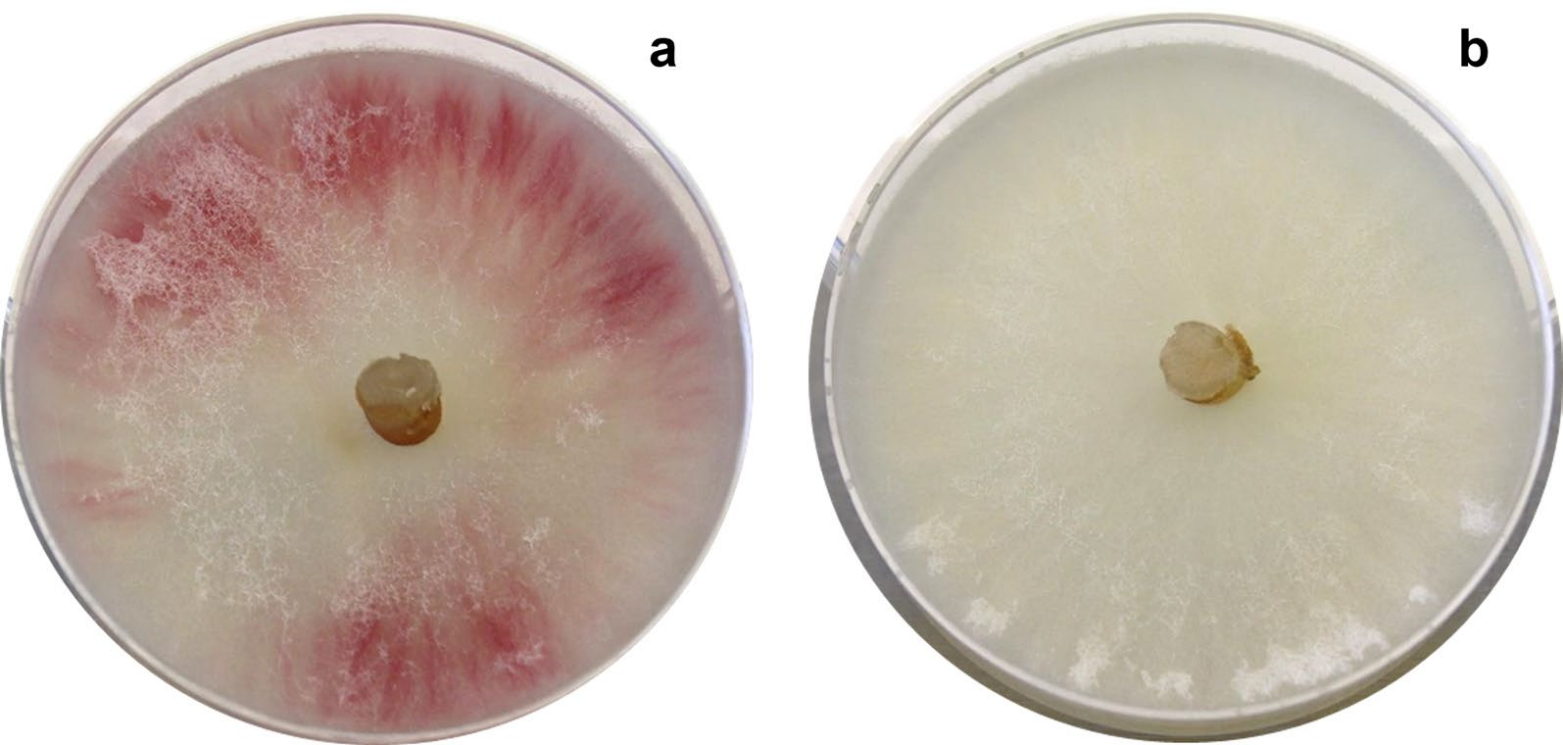
Phenotypes on potato sucrose agar of *F. venenatum *A3/5 (**a**) and *PKS12 *gene CRISPR/Cas9 variants (**b**)

From each of the viable colonies recovered, at least 7 single spore isolates were cultured on non-selective PSA plates for phenotypic screening (Fig. 2). Albino phenotypes, indicative of non-functional PKS12 protein [21] were obtained from all single spore isolates of pFC332::5S-PK3/14 transformed protoplasts, but only 30–40% of isolates, of just 3 of the 11 colonies from pFC332::PolII-PK3/14 transformed protoplasts (Table 3). Sequence data from target site amplicons show a single adenine base insertion at position ^−^1 of the cut site in albino variants (Additional file 1: Table S1), except isolates from colony 5–7 from pFC332::PolII-PK3/14 transformed protoplasts for which no evidence of target site indels was obtained.

**Table 3:** Phenotypes on PSA of isolates from putative *PKS12* gene variants

		Phenotype		
Putative variant	Number of isolates	Red	Albino	% albino phenotypes
pFC332 control	4	4	–	0
5S-1	7	–	7	100
5S-2	7	–	7	100
5S-4	10	–	10	100
5S-5	9	–	9	100
5S-6	10	–	10	100
5S-7	10	–	10	100
5S-10	10	–	10	100
PolII-1	12	12	–	0
PolII-2	9	9	–	0
PolII-3	9	9	–	0
PolII-4	14	14	–	0
PolII-5	10	7	3	30
PolII-6	10	6	4	40
PolII-7	11	7	4	36
PolII-8	10	10	–	0
PolII-9	10	9	–	0
PolII-12	10	9	–	0
PolII-13	10	10	–	0

Putative *PKS12* gene variants were generated using sgRNAs transcribed from the PolIII promoter *PFv5SrRNA* (5S) or the PolII promoter *PgdpA* (PolII). Control vector pFC332 is without sgRNA constructs. Isogenic isolates were cultured on potato sucrose agar (PSA), which induces red pigmentation in strains with functional polyketide synthase encoded by the *PKS12* gene. *PKS12* frameshift variants have an albino phenotype.

## Discussion

This is the first report of CRISPR/Cas mediated gene editing in the industrially important fungus *F. venenatum*, used in production of mycoprotein. We demonstrate efficient transgene-free target site mutation of the endogenous marker gene *PKS12* by transient expression of CRISPR constituents from an AMA1 replicator vector, use of which has been reported for other fungi but not the genus *Fusarium*.

Use of an autonomously replicating vector for transient expression of CRISPR reagents and selection markers avoids the difficulties associated with *in-vitro* generated unstable sgRNAs, making this an attractive system for generating transgene-free improved strains of *F. venenatum* and enables use of the same selection marker for successive gene editing Most isolates obtained were unable to grown on hygromycin after a period of culture on non-selective medium (Additional file 2: Table S2), indicating loss of the AMA1 vector, and the continuing hygromycin resistance observed in a small number of isolates suggests either possible chromosomal integration of vector elements (including the *hph* gene for hygromycin resistance), or residual extrachromosomal vector in some nuclei (which may be due to higher initial copy number within some isolates). Eventual loss from all nuclei would be expected through continued culture in non-selective conditions, as reported by Leeuwe et al. [22], or after repeated single spore isolation [7].

High efficiency editing is a prerequisite for enabling low input screening of transformants to identify target site variants and may be achieved by optimising levels of sgRNA transcription and nuclear-localised Cas9. Efficiencies of editing in filamentous fungi have been shown to vary depending on the promoter driving sgRNA transcription and between different species and experimental systems, e.g. Nodvig et al.[7, 20] speculate that differences in gene editing efficiency observed in *Aspergillus* may depend on different requirements for *cas9* codon usage and levels of *cas9* and sgRNA, which may vary in correlation with AMA1 vector copy number and also promoter, reporting higher efficiencies from sgRNAs expressed from an endogenous PolIII *U3* promoter compared to the polymerase II/ribozyme-based system. Likewise, Shi et al.[16] reported enhanced gene editing in *F. fujikuroi* using promoters for highly expressed PolIII endogenous genes, but they were unsuccessful using the heterologous PolII (*PgdpA*) dual ribozyme system. We demonstrate similar results in *F. venenatum* using an AMA1 vector for in vivo production of CRISPR reagents, achieving edits in 100% of recovered isolates when sgRNAs were transcribed from the native *Fv5SrRNA* promoter and greatly reduced efficacy using the *PgdpA* ribozyme system, and RT-qPCR results from our preliminary experiments (Additional file 3: Figure S1, Table S3) suggest relatively low levels of transcription from *PgdpA* in AMA1 vector transformants.

The importance of the NLS in determining efficiency of Cas9 protein nuclear import and subsequent gene editing success has also been highlighted by several studies. SV40_NLS_ has been used widely for Cas9 nuclear import in eukaryotes including other filamentous fungi [7, 8, 12, 20]; however in *Fusarium*, Wang et al.[14] and Shi et al.[16] were unsuccessful in obtaining gene edits using Cas9-SV40NLS delivered as a constituent of a RNP or transcribed from an expression vector (but were successful using a Cas9-HBT_NLS_ protein). In our experiments, we were successful in obtaining gene edits using *cas9-*SV40_NLS_ transcripts, possibly as relatively high levels of transcription can be obtained from genes driven by the *tef1* promoter, when expressed from an autonomously replicating vector (Additional file 3: Figure S1, Table S3).

Cas9 optimization is another key factor reported to influence gene editing success in filamentous fungi, and differences in reported effectiveness and toxicity may be ascribed to codon usage as well as potential differences in levels of nuclear Cas9 resulting from the different methodologies employed. Foster et al.[12] reported toxicity of Cas9 optimized for both *N. crassa* and the target organism in *M. oryzae* when *cas9* was expressed constitutively from integrated transgenes but they were able to recover gene edited mutants using hSpCas9 delivered transiently as RNPs. Fuller et al.[6] found no evidence of toxicity from hSpCas9 in *Aspergillus* transgenic lines, likewise Shi et al. [16] observed no effect on growth of *F. fujikuroi* of optimised *FuCas9* using expression vectors, and in *F. graminearum* Gardiner and Kazan [13] were successful with *F. graminearum* optimised Cas9 produced in transgenic lines. However, in our experiments using an AMA1 vector for transcription of *cas9* using the *tef1* promoter we were unable to recover viable colonies from protoplasts expressing Cas9 optimised for *F. venenatum* and recovery rates were low after transformation with vectors expressing *A. niger* Cas9, which is indicative of potential Cas9 toxicity (see also Additional file 4: Table S4). Additionally, our results show reduced viability of protoplasts and mycelium expressing *PFv5SrRNA*-sgRNAs compared to those expressing *PgdpA*-sgRNAs, suggesting a possible interaction between sgRNA transcription levels and Cas9 toxicity.

## Conclusions

In conclusion, we have demonstrated 100% gene editing frequency in isolates of *F. venenatum* by transforming protoplasts with AMA1 replicator vectors expressing *A. niger* codon-optimised *cas9-*SV40_NLS_ transcribed from the *tef1* promoter and sgRNAs transcribed from the endogenous *Fv5SrRNA* promoter. Evidence of AMA1 vector loss in the absence of selection makes this an attractive system for generating transgene-free gene edited strains of *F. venenatum* and for performing successive gene edits using the same selection marker. However, reduced protoplast viability due to potential Cas9 toxicity was observed, which may impede development of further CRISPR/Cas technologies such as gene insertion, base editing and prime editing in *F. venenatum*. Assessment of the efficacy of alternative promoters and/or use of an inducible system for regulating *cas9* expression would enable optimization of Cas9 production and allow re-evaluation of Cas9 codon-optimized for *F. venenatum*, which was apparently toxic when expressed from the *tef1* promoter using an AMA1 vector. Although each of the elements for gene editing reported here have been described previously in fungi, such as use of AMA1 vectors for delivery of CRISPR constituents and use of PolII-ribozyme and 5SrRNA promotors for driving sgRNA expression, this is the first report of using an AMA1 vector for gene editing in *Fusarium* and the findings reported here should also be of interest more widely for transgene-free gene editing in fungi. Implementation of the gene editing methodology described here, and development of additional CRISPR/Cas technologies will further an understanding of the controls governing growth and metabolite synthesis in *F. venenatum*, will aid identification of targets for strain improvement using traditional methods and will facilitate innovation in fermentation development.

## Methods

### Vector construction

Vector backbones were obtained by restriction digest and component parts for vector inserts were generated by PCR from synthesised DNA or existing vector templates using Q5® High-Fidelity 2X Master Mix (NEB). Vector components were purified using the Monarch® DNA Gel Extraction Kit or the Monarch® PCR & DNA Cleanup Kit (NEB) and assemblies were performed using NEBuilder® HiFi DNA Assembly Master Mix (NEB). For details of primers used in vector construction see Additional file 5: Table S5. Recombinant vectors were recovered by transformation of NEB® 10-beta Competent E. coli (High Efficiency) cells and subsequent plasmid isolation was performed using the NucleoSpin Plasmid kit (Macherey–Nagel). For comparison of *Fv* Cas9 and *A. niger* Cas 9 a codon-optimised *FvCas9* gene was synthesised and an expression cassette Ptef1-Fvcas9-SV40-Ttef1 was assembled (Additional file 6: Table S6) and ligated with the backbone of AMA1 vector pFC332 [7], replacing the *A. niger cas9* cassette to give vector pFCFvCas9. These vectors were used as ‘empty vector’ controls in transformation experiments. The sequence for the target site (‘14’) in exon 3 of the endogenous *PKS12* gene was cloned downstream of promoter *PgdpA* within a dual ribozyme cassette [7] (giving construct PolII-PK3/14) or promoter *PFv5SrRNA* (giving construct 5S-PK3/14) (Fig. 1, and see Additional file 7: Table S7 for *PFv5SrRNA* cassette sequence). Promoter-sgRNA constructs were cloned into the USER site of pFC332 to give single sgRNA vectors pFC332::PolII-PK3/14 and pFC332::5S-PK3/14. For comparison of gene editing efficiency with *Fvcas9*, PolII-PK3/14 was additionally cloned into the USER site of vector pFCFvCas9 to give pFCFvCas9::PolII-PK3/14.

Details of vector components and assembly are as follows: codon usage preference of predicted highly expressed genes for *F. venenatum* A3/5 was determined (Additional file 8: Table S8) and used to generate a codon-optimised sequence for *cas9*. Using Optimizer [23] ‘Relative Synonymous Codon Usage’ (RSCU) was performed, selecting options for ‘standard genetic code’ and ‘one amino acid—one codon’. The coding sequence was subsequently domesticated to enable use in Golden Gate cloning if required and DNA for the resulting sequence, (Additional file 6: Table S6), was synthesised (GeneArt, Invitrogen). The AMA1 replication vector backbone (pFCBB) of 10,129 bases was obtained from pFC332 [7] by restriction digest using PacI and BamHI-HF® (NEB), excising part of the Nt.BbvCI–PacI cloning site, the *tef1* promoter, *cas9* coding sequence and 489 bases (5’-3’) of the *tef1* terminator. The residual *tef1* terminator (87 bases) was incorporated in recombinant vector assembly by overlap with inserts terminated with *Ttef1*. The SV40_NLS_-stop codon, *tef1* terminator and *tef1* promoter parts were PCR-amplified from template pFC332. *FvCas9* was PCR amplified in one part from synthesised DNA and assembled with the *tef1* promoter and *tef1* terminator parts in Golden Gate vector pICH47751 [24] to give Ptef1-FvCas9-SV40-Ttef1. This was used as a template for generation by PCR of two parts for assembly with pFCBB to give pFCFvCas9: *Ptef1* and *cas9* bases 1–2141 (5’-3’) were amplified as Part 1 using primers NtPac1_Ptef1 (pFCBB)_F and FvCas9-1_R; and Part 2 was generated by primers FvCas9-2_F and Ttef1_R_no ext, incorporating *cas9* bases 2142–4376 (5’-3’) and sequences for SV40-stop codon and *Ttef1*. The Nt.BbvCI –PacI cloning site was reconstructed by incorporating required bases into the 5’ extension of primer NtPac1_Ptef1 (pFCBB)_F. The PolII/ribozyme sgRNA cassettes were constructed using pFC334 as a template and assembled into the Nt.BbvCI-PacI USER site of pFC332 as described by Nodvig et al.[7]. *5SrRNA* sgRNA cassettes were synthesised by IDT (Additional file 7: Table S7) and PCR amplified using primers 5SrRNA_F USER and 5SrRNA_R USER for USER assembly into pFC332 or pFCBB. Details of USER reactions are described in Additional file 9: Table S9.

### Protoplast isolation and transformation

A method modified from Moradi et al*.* [25] was used for protoplast transformation. Media are detailed in Additional file 10: Table S10. To obtain approximately 40 × 100 µl protoplast aliquots, 250 ml CMC medium in a 500 ml Erlenmeyer flask was inoculated with 5 × 4 mm plugs from an agar plate culture and incubated for 6 days on a rotary shaker at 25 °C, shaking at 175 rpm (or to give good aeration). The culture was filtered through 2 layers of sterile Miracloth (Millipore) and the filtrate was centrifuged at 4000×*g* for 10 min. After decanting the supernatant, the conidia were resuspended in 100 ml of YEPD broth in a 250 ml Erlenmeyer flask and incubated overnight (14 h) in a rotary shaker at 22 °C, shaking at 175 rpm. The mycelium was harvested by filtration using 2 layers of sterile Miracloth and was rinsed with sterile pure water. Excess water was gently removed by blotting, without compacting the mycelium, and the mycelial mat was transferred to 20 ml Protoplasting Buffer in a sterile beaker and gently dispersed to ensure efficient digestion. After 2 h incubation at 28 °C, 80 rpm, the digestion mixture was filtered through sterile Miracloth (3 layers) into 50 ml sterile conical bottomed plastic tubes. The filtrate was centrifuged at RT at 3000×*g* for 5 min, the supernatant was immediately decanted, and the protoplasts were washed: using a 25 ml sterile filtered serological pipette the pellet was gently dislodged and resuspended (using both manual and air agitation) in 30 ml of STC, before centrifuging at 3,000 × g for 5 min. The wash was repeated (removing an aliquot of the second suspension for protoplast quantification) and the protoplasts were resuspended in STC Buffer to give 1 × 10^8^ protoplasts/ml before dispensing 100 µl aliquots into sterile 2 ml microcentrifuge tubes. For protoplast transformation 1–5 µg DNA was added and the tube was flicked gently 3 times to mix before incubating at RT for 20 min. 1 ml 40% PTC was then added, and the tube was inverted gently three times to mix and incubated at RT for 20 min before decanting into a 50 ml sterile tube containing 5 ml TB3 supplemented with 100 µg ml^−1^ Hygromycin B and incubated at 21 °C, shaking at 90 rpm for 14–16 h (overnight). Each sample was decanted into a 9 cm petri dish before adding 10 ml Top Agar (cooled to ‘hand hot’ and kept molten in a 50 °C water bath), supplemented with Hygromycin B to give 100 µg ml^−1^ (one plate was included with no selection for a non-transformed protoplast control), swirling gently to mix. Plates were incubated for 8–10 h at RT before adding 10 ml Top Agar prepared as before and supplemented with 100 µg ml^−1^ Hygromycin B (except for the control), sealed and incubated at 20 °C. Colonies from transformation plates were transferred after 10 days to PDA supplemented with Hygromycin B (75 µg ml^−1^) for maintenance of the AMA1 vector, then cultured for 2 weeks at 28 °C. For isolation of isogenic lines, agar plugs from colonies were inoculated in 20 ml CMC medium in a 100 ml Erlenmeyer flask and incubated shaking at 175 rpm for up to 7 days at 25 °C. Spores were collected by filtration through 2 layers of Miracloth, pelleted at 4000xg for 10 min and plated to give a low density on PDA plates. After incubation for 14–15 h at 20 °C single germinating spores were isolated and incubated in the dark at 28 °C on PSA (Additional file 11: Table S11) for identifying albino *PKS12* variants.

### CRISPR variant analysis

Genomic DNA for use in PCR was extracted from mycelium cultured in 50 ml plastic conical based tubes containing 10 ml of a proprietary medium and incubated shaking at 175 rpm for 5–7 days at 28 °C. The culture was collected on Miracloth, rinsed with autoclaved purified water, blotted to remove excess liquid and added with 2 ball bearings (4 mm chrome steel) to 2 ml microcentrifuge tubes before flash freezing in liquid nitrogen and storage at −80 °C. Mycelium was ground while frozen using a ball mill (Retsch) and DNA was extracted using the DNeasy Plant Mini Kit (Qiagen). PCR amplicons were generated using Q5® High-Fidelity 2X Master Mix (NEB) using primers PKS12_F2 and PKS12_R2, which span the target region (Fig. 1). Amplicons were Sanger sequenced (Eurofins Genomics) using sequencing primers PKS12-Seq_R1 and PKS12-Seq_F2.

### Sequencing analysis and software

Nucleotide and protein alignments were performed using Geneious version 10.0.2 (http://www.geneious.com) [26]. Schematic maps were prepared using IBS software [27].

## Supplementary Information

**Additional file 1: Table S1.** *PKS12* gene target site sequence data for phenotypic variants.

**Additional file 2: Table S2.** Persistence of hygromycin tolerance in CRISPR variants.

**Additional file 3: Figure S1.** RT-qPCR analysis of *F. venenatum* AMA1 vector *cas9* transcripts expressed in *F. venenatum*; **Table S3.** Primers used in RT-qPCR analysis.

**Additional file 4: Table S4** Viability of protoplasts transformed with AMA1 vectors expressing *mEGFP* and *cas9*.

**Additional file 5: Table S5**. Primers used in vector construction and PCR analysis of PKS12 gene variants.

**Additional file 6: Table S6.** Expression cassette Ptef1-FvCas9-SV40-Ttef1 used in this study.

**Additional file 7: Table S7.** Sequence of *PFv5SrRNA*-sgRNA cassettes used in this study.

**Additional file 8: Table S8.** Codon usage table for highly expressed genes in *F. venenatum*.

**Additional file 9: Table S9.** Method for USER cloning of promoter-sgRNA cassettes into AMA1 vectors.

**Additional file 10: Table S10**. Media used for making protoplasts and protoplast transformation.

**Additional file 11: Table S11.** Method for making Potato Sucrose Agar (PSA).

## Abbreviations

Cas9: CRISPR associated DNA-cutting enzyme
CRISPR: Clustered regularly interspaced short palindromic repeats
HDV ribozyme: Hepatitis Delta Virus ribozyme
HH ribozyme: Hammerhead ribozyme
indel: Insertion-deletion mutation of genomic sequence
Kb: Kilobase pairs
mEGFP: Monomeric enhanced green fluorescent protein
mRFP: Monomeric red fluorescent protein
PDA: Potato Dextrose Agar
PolII: Polymerase II
PolIII: Polymerase III
USER: Uracil-Specific Excision Reagent
PCR: Polymerase chain reaction
RT-qPCR: Reverse transcription quantitative PCR
