# Supplementary material for: CRISPR/Cas9 mediated editing of the Quorn fungus Fusarium venenatum A3/5 by transient expression of Cas9 and sgRNAs targeting endogenous marker gene PKS12
Source: Fungal Biol Biotechnol. 2021 Nov 17;8:15. doi: 10.1186/s40694-021-00121-8 (PMC8597179; doi:10.1186/s40694-021-00121-8)
Supplement: Supplementary file 2 — Additional file 2: Table S2. Persistence of hygromycin tolerance in CRISPR variants. [file 40694_2021_121_MOESM2_ESM.docx]

**Additional File 2**

**Table S2**  **Persistence of hygromycin tolerance in CRISPR variants**

| CRISPR variant | Growth on PDA | Growth on PDA+Hyg |
| --- | --- | --- |
| 5S-1 | 3 | 0 |
| 5S-2 | 3 | 0 |
| 5S-4 | 3 | 0 |
| 5S-5 | 3 | 0 |
| 5S-6 | 3 | 3 |
| 5S-7 | 3 | 2 |
| 5S-10 | 3 | 3 |
| PolII-5 | 3 | 0 |
| PolII-6 | 3 | 0 |
| PolII-7 | 3 | 0 |
| Control cultures | | |
| AMA1-A | y | y |
| AMA1-B | y | y |
| WT | y | x |

Viability (growth) on hygromycin (selective) PDA (PDA+Hyg) of isogenic lines from CRISPR *PKS12* gene variants using inoculum from cultures previously maintained on non-selective media for two culture passages. Results are shown for 3 isogenic lines from each variant. Variants were generated using sgRNAs transcribed from the PolIII promoter *PFv5SrRNA* (5S) or the PolII promoter *PgdpA* (PolII) respectively. Control cultures transformed with AMA1 vectors expressing *mEGFP* (AMA1-A and AMA1-B) were maintained on selection medium for several culture passages and growth for three replicates of each is denoted by y (growth) or x (no growth).
