# Supplementary material for: CRISPR/Cas9 mediated editing of the Quorn fungus Fusarium venenatum A3/5 by transient expression of Cas9 and sgRNAs targeting endogenous marker gene PKS12
Source: Fungal Biol Biotechnol. 2021 Nov 17;8:15. doi: 10.1186/s40694-021-00121-8 (PMC8597179; doi:10.1186/s40694-021-00121-8)
Supplement: Supplementary file 4 — Additional file 4: Table S4 Viability of protoplasts transformed with AMA1 vectors expressing mEGFP and cas9. [file 40694_2021_121_MOESM4_ESM.docx]

**Additional File 4**

**Table S4 Viability of protoplasts transformed with AMA1 vectors expressing *mEGFP* and *cas9***

| Protoplast-derived colonies | Selection plate |
| --- | --- |
| A - pFC::PgpdA-mEGFP-SV40-tTrpC | 110 |
| B - pFC332:: PgpdA-mEGFP-SV40-tTrpC (Cas9) | 7 |

Number of colonies observed on hygromycin selection plates following transformation of protoplasts with AMA1 vectors expressing *mEGFP* (A) or *mEGFP* and *A. niger cas9* (B).
