# Supplementary material for: CRISPR/Cas9 mediated editing of the Quorn fungus Fusarium venenatum A3/5 by transient expression of Cas9 and sgRNAs targeting endogenous marker gene PKS12
Source: Fungal Biol Biotechnol. 2021 Nov 17;8:15. doi: 10.1186/s40694-021-00121-8 (PMC8597179; doi:10.1186/s40694-021-00121-8)
Supplement: Supplementary file 6 — Additional file 6: Table S6. Expression cassette Ptef1-FvCas9-SV40-Ttef1 used in this study. [file 40694_2021_121_MOESM6_ESM.docx]

**Additional File 6**

**Table S6 Expression cassette *Ptef1-FvCas9-SV40-Ttef1* used in this study**

CGAGACAGCAGAATCACCGCCCAAGTTAAGCCTTTGTGCTGATCATGCTCTCGAACGGGCCAAGTTCGGGAAAAGCAAAGGAGCGTTTAGTGAGGGGCAATTTGACTCACCTCCCAGGCAACAGATGAGGGGGGCAAAAAGAAAGAAATTTTCGTGAGTCAATATGGATTCCGAGCATCATTTTCTTGCGGTCTATCTTGCTACGTATGTTGATCTTGACGCTGTGGATCAAGCAACGCCACTCGCTCGCTCCATCGCAGGCTGGTCGCAGACAAATTAAAAGGCGGCAAACTCGTACAGCCGCGGGGTTGTCCGCTGCAAAGTACAGAGTGATAAAAGCCGCCATGCGACCATCAACGCGTTGATGCCCAGCTTTTTCGATCCGAGAATCCACCGTAGAGGCGATAGCAAGTAAAGAAAAGCTAAACAAAAAAAAATTTCTGCCCCTAAGCCATGAAAACGAGATGGGGTGGAGCAGAACCAAGGAAAGAGTCGCGCTGGGCTGCCGTTCCGGAAGGTGTTGTAAAGGCTCGACGCCCAAGGTGGGAGTCTAGGAGAAGAATTTGCATCGGGAGTGGGGCGGGTTACCCCTCCATATCCAATGACAGATATCTACCAGCCAAGGGTTTGAGCCCGCCCGCTTAGTCGTCGTCCTCGCTTGCCCCTCCATAAAAGGATTTCCCCTCCCCCTCCCACAAAATTTTCTTTCCCTTCCTCTCCTTGTCCGCTTCAGTACGTATATCTTCCCTTCCCTCGCTTCTCTCCTCCATCCTTCTTTCATCCATCTCCTGCTAACTTCTCTGCTCAGCACCTCTACGCATTACTAGCCGTAGTATCTGAGCACTTCTCCCTTTTATATTCCACAAAACATAACACAACCTTCACCATGGACAAGAAGTACTCTATCGGCCTCGACATCGGCACCAACTCTGTCGGCTGGGCTGTCATCACCGACGAGTACAAGGTCCCTTCTAAGAAGTTCAAGGTCCTCGGCAACACCGACCGACACTCTATCAAGAAGAACCTCATCGGCGCTCTCCTCTTCGACTCTGGCGAaACCGCTGAGGCTACCCGACTCAAGCGAACCGCTCGACGACGATACACCCGACGAAAGAACCGAATCTGCTACCTCCAGGAGATCTTCTCTAACGAGATGGCTAAGGTCGACGACTCTTTCTTCCACCGACTCGAGGAGTCTTTCCTCGTCGAGGAGGACAAGAAGCACGAGCGACACCCTATCTTCGGCAACATCGTCGACGAGGTCGCTTACCACGAGAAGTACCCTACCATCTACCACCTCCGAAAGAAGCTCGTCGACTCTACCGACAAGGCTGACCTCCGACTCATCTACCTCGCTCTCGCTCACATGATCAAGTTCCGAGGCCACTTCCTCATCGAGGGCGACCTCAACCCTGACAACTCTGACGTCGACAAGCTCTTCATCCAGCTCGTCCAGACCTACAACCAGCTCTTCGAGGAGAACCCTATCAACGCTTCTGGCGTCGACGCTAAGGCTATCCTCTCTGCTCGACTCTCTAAGTCTCGACGACTCGAGAACCTCATCGCTCAGCTCCCTGGCGAGAAGAAGAACGGCCTCTTCGGCAACCTCATCGCTCTCTCTCTCGGCCTCACCCCTAACTTCAAGTCTAACTTCGACCTCGCTGAGGACGCTAAGCTCCAGCTCTCTAAGGACACCTACGACGACGACCTCGACAACCTCCTCGCTCAGATCGGCGACCAGTACGCTGACCTCTTCCTCGCTGCTAAGAACCTCTCTGACGCTATCCTCCTCTCTGACATCCTCCGAGTCAACACCGAGATCACCAAGGCTCCTCTCTCTGCTTCTATGATCAAGCGATACGACGAGCACCACCAGGACCTCACCCTCCTCAAGGCTCTCGTCCGACAGCAGCTCCCTGAGAAGTACAAGGAGATCTTCTTCGACCAGTCTAAGAACGGCTACGCTGGCTACATCGACGGCGGCGCTTCTCAGGAGGAGTTCTACAAGTTCATCAAGCCTATCCTCGAGAAGATGGACGGCACCGAGGAGCTCCTCGTCAAGCTCAACCGAGAGGACCTCCTCCGAAAGCAGCGAACCTTCGACAACGGCTCTATCCCTCACCAGATCCACCTCGGCGAGCTCCACGCTATCCTCCGACGACAGGAGGACTTCTACCCTTTCCTCAAGGACAACCGAGAGAAGATCGAGAAGATCCTCACCTTCCGAATCCCTTACTACGTCGGCCCTCTCGCTCGAGGCAACTCTCGATTCGCTTGGATGACCCGAAAGTCTGAGGAaACCATCACCCCTTGGAACTTCGAGGAGGTCGTCGACAAGGGCGCTTCTGCTCAGTCTTTCATCGAGCGAATGACCAACTTCGACAAGAACCTCCCTAACGAGAAGGTCCTCCCTAAGCACTCTCTCCTCTACGAGTACTTCACCGTCTACAACGAGCTCACCAAGGTCAAGTACGTCACCGAGGGCATGCGAAAGCCTGCTTTCCTCTCTGGCGAGCAGAAGAAGGCTATCGTCGACCTCCTCTTCAAGACCAACCGAAAGGTCACCGTCAAGCAGCTCAAGGAGGACTACTTCAAGAAGATCGAGTGCTTCGACTCTGTCGAGATCTCTGGCGTCGAGGACCGATTCAACGCTTCTCTCGGCACCTACCACGACCTCCTCAAGATCATCAAGGACAAGGACTTCCTCGACAACGAGGAGAACGAGGACATCCTCGAGGACATCGTCCTCACCCTCACCCTCTTCGAGGACCGAGAGATGATCGAGGAGCGACTCAAGACCTACGCTCACCTCTTCGACGACAAGGTCATGAAGCAGCTCAAGCGACGACGATACACCGGCTGGGGCCGACTCTCTCGAAAGCTCATCAACGGCATCCGAGACAAGCAGTCTGGCAAGACCATCCTCGACTTCCTCAAGTCTGACGGCTTCGCTAACCGAAACTTCATGCAGCTCATCCACGACGACTCTCTCACCTTCAAGGAGGACATCCAGAAGGCTCAGGTtTCTGGCCAGGGCGACTCTCTCCACGAGCACATCGCTAACCTCGCTGGCTCTCCTGCTATCAAGAAGGGCATCCTCCAGACCGTCAAGGTCGTCGACGAGCTCGTCAAGGTCATGGGCCGACACAAGCCTGAGAACATCGTCATCGAGATGGCTCGAGAGAACCAGACCACCCAGAAGGGCCAGAAGAACTCTCGAGAGCGAATGAAGCGAATCGAGGAGGGCATCAAGGAGCTCGGCTCTCAGATCCTCAAGGAGCACCCTGTCGAGAACACCCAGCTCCAGAACGAGAAGCTCTACCTCTACTACCTCCAGAACGGCCGAGACATGTACGTCGACCAGGAGCTCGACATCAACCGACTCTCTGACTACGACGTCGACCACATCGTCCCTCAGTCTTTCCTCAAGGACGACTCTATCGACAACAAGGTCCTCACCCGATCTGACAAGAACCGAGGCAAGTCTGACAACGTCCCTTCTGAGGAGGTCGTCAAGAAGATGAAGAACTACTGGCGACAGCTCCTCAACGCTAAGCTCATCACCCAGCGAAAGTTCGACAACCTCACCAAGGCTGAGCGAGGCGGCCTCTCTGAGCTCGACAAGGCTGGCTTCATCAAGCGACAGCTCGTCGAaACCCGACAGATCACCAAGCACGTCGCTCAGATCCTCGACTCTCGAATGAACACCAAGTACGACGAGAACGACAAGCTCATCCGAGAGGTCAAGGTCATCACCCTCAAGTCTAAGCTCGTtTCTGACTTCCGAAAGGACTTCCAGTTCTACAAGGTCCGAGAGATCAACAACTACCACCACGCTCACGACGCTTACCTCAACGCTGTCGTCGGCACCGCTCTCATCAAGAAGTACCCTAAGCTCGAGTCTGAGTTCGTCTACGGCGACTACAAGGTCTACGACGTCCGAAAGATGATCGCTAAGTCTGAGCAGGAGATCGGCAAGGCTACCGCTAAGTACTTCTTCTACTCTAACATCATGAACTTCTTCAAGACCGAGATCACCCTCGCTAACGGCGAGATCCGAAAGCGACCTCTCATCGAaACCAACGGCGAaACCGGCGAGATCGTCTGGGACAAGGGCCGAGACTTCGCTACCGTCCGAAAGGTCCTCTCTATGCCTCAGGTCAACATCGTCAAGAAaACCGAGGTCCAGACCGGCGGCTTCTCTAAGGAGTCTATCCTCCCTAAGCGAAACTCTGACAAGCTCATCGCTCGAAAGAAGGACTGGGACCCTAAGAAGTACGGCGGCTTCGACTCTCCTACCGTCGCTTACTCTGTCCTCGTCGTCGCTAAGGTCGAGAAGGGCAAGTCTAAGAAGCTCAAGTCTGTCAAGGAGCTCCTCGGCATCACCATCATGGAGCGATCTTCTTTCGAGAAGAACCCTATCGACTTCCTCGAGGCTAAGGGCTACAAGGAGGTCAAGAAGGACCTCATCATCAAGCTCCCTAAGTACTCTCTCTTCGAGCTCGAGAACGGCCGAAAGCGAATGCTCGCTTCTGCTGGCGAGCTCCAGAAGGGCAACGAGCTCGCTCTCCCTTCTAAGTACGTCAACTTCCTCTACCTCGCTTCTCACTACGAGAAGCTCAAGGGCTCTCCTGAGGACAACGAGCAGAAGCAGCTCTTCGTCGAGCAGCACAAGCACTACCTCGACGAGATCATCGAGCAGATCTCTGAGTTCTCTAAGCGAGTCATCCTCGCTGACGCTAACCTCGACAAGGTCCTCTCTGCTTACAACAAGCACCGAGACAAGCCTATCCGAGAGCAGGCTGAGAACATCATCCACCTCTTCACCCTCACCAACCTCGGCGCTCCTGCTGCTTTCAAGTACTTCGACACCACCATCGACCGAAAGCGATACACCTCTACCAAGGAGGTCCTCGACGCTACCCTCATCCACCAGTCTATCACCGGCCTCTACGAaACCCGAATCGACCTCTCTCAGCTCGGCGGCGACTGA***TG*CCTCCCAAGAAGAAGCGCAAGGTCTGAGCGGACATTCGATTTATGCCGTTATGACTTCCTTAAAAAAGCCTTTACGAATGAAAGAAATGGAATTAGACTTGTTATGTAGTTGATTCTACAATGGATTATGATTCCTGAACTTCAAATCCGCTGTTCATTATTAATCTCAGCTCTTCCCGTAAAGCCAATGTTGAAACTATTCGTAAATGTACCTCGTTTTGCGTGTACCTTGCTTATCACGTGATATTACATGACCTGGACAGAGTTCTGCGCGAAAGTCATAACGTAAATCCCGGGCGGTAGGTGCGTCCCGGGCGGAAGGTAGTTTTCTCGTCCACCCCAACGCGTTTATCAACCTCAACTTTCAACAACCATCATGCCACCAAAAGCGCGTAAAACAAAGCGAGATTTGATTGAGCAAGAGGGCAGGATCCAATGCGCGATTCAAGACATTAAAAATGGAAAATTTCAAAAAATTGCGCCCGCAGCGCGTGCATACAAAATTCATCCCAATAC**

Constituent parts of the *Ptef1-FvCas9-SV40-Ttef1* cassette are denoted by different font colours: red font is sequence for *Ptef1*; Black font is coding sequence for the *Cas9* gene codon-optimised for Fusarium venenatum (*Fv_Cas9*) used in this study; pink bold font is sequence for SV40_NLS_-STOP codon (preceding bases in italics were added for frameshift) ; blue bold font is sequence for *Ttef1*.
