# Supplementary material for: CRISPR/Cas9 mediated editing of the Quorn fungus Fusarium venenatum A3/5 by transient expression of Cas9 and sgRNAs targeting endogenous marker gene PKS12
Source: Fungal Biol Biotechnol. 2021 Nov 17;8:15. doi: 10.1186/s40694-021-00121-8 (PMC8597179; doi:10.1186/s40694-021-00121-8)
Supplement: Supplementary file 7 — Additional file 7: Table S7. Sequence of PFv5SrRNA-sgRNA cassettes used in this study. [file 40694_2021_121_MOESM7_ESM.docx]

**Additional File 7**

**Table S7 Sequence of *P5SrRNA*-sgRNA cassettes used in this study**

*P5SrRNA* sgRNA cassettes CACATACGACCAAAGGTAGTGGAAAATACGGGATCCCGTCCGCTCTCCCATAGTCAAGCCACTAACCGGCGGATTAGTAGTTGGGTCGGTGACGACCAGCGAATCCCCGCTGTTGTATGTNNNNNNNNNNNNNNNNNNNNGTTTTAGAGCTAGAAATAGCAAGTTAAAATAAGGCTAGTCCGTTATCAACTTGAAAAAGTGGCACCGAGTCGGTGC**TTTTTT***GTAGTAACACCCGGAGTGCATGGATCA*

Capital font = 5SRNA promoter sequence; ‘N’s = *PKS12* gene target site sequence PK3-14 (CCAGAACAGCCTGAAATGAG); underlined capital font = scaffold sequence; bold font = terminator sequence; capital italics = spacer sequence
