# Supplementary material for: CRISPR/Cas9 mediated editing of the Quorn fungus Fusarium venenatum A3/5 by transient expression of Cas9 and sgRNAs targeting endogenous marker gene PKS12
Source: Fungal Biol Biotechnol. 2021 Nov 17;8:15. doi: 10.1186/s40694-021-00121-8 (PMC8597179; doi:10.1186/s40694-021-00121-8)
Supplement: Supplementary file 9 — Additional file 9: Table S9. Method for USER cloning of promoter-sgRNA cassettes into AMA1 vectors. [file 40694_2021_121_MOESM9_ESM.docx]

**Additional File 9**

**Table S9 Method for USER cloning of promoter-sgRNA cassettes into AMA1 vectors**

| *PCR amplification of inserts* |
| --- |
| In a total volume of 50 µl: |
| 1X Phusion™ High-Fidelity DNA Polymerase Buffer |
| 200 µM dNTPs |
| 0.5 µM each primer (F & R) |
| 2 pg template |
| Phusion™ High-Fidelity DNA Polymerase (Thermo Fisher Scientific) 0.5 units. |
| PCR cycle |
| 98 °C 30s x1 cycle |
| 98 °C 10s, ‘x’ °C 30s |
| 72 °C 15s per Kb x35 cycles |
| 72 °C 5 min x1 cycle |
| PCR products were gel purified, eluted in water and quantified by Qubit™ assay |
| *USER reactions* |
| Final concentrations of components in a total volume of 12 µl: |
| 50 ng vector linearised and gel purified. |
| 1.5 mM MgCl_2_; |
| 1X Applied Biosystems™ PCR Buffer II without MgCl_2_ |
| USER® enzyme 1.2 units |
| Purified inserts (eluted in water): for PolII/ribozyme cassettes use equimolar insert:vector ratio;  for 5SRNA cassettes use 5:1 molar insert:vector ratio |
| Water to a final volume of 12 µl |
| Incubate at 37 °C 25 minutes, 25 °C 25 minutes |
| *Recovery of recombinant vectors* |
| Add entire reaction to 50 µl chemically competent E. coli. Follow protocol provided with cells |
