# Supplementary material for: CRISPR/Cas9 mediated editing of the Quorn fungus Fusarium venenatum A3/5 by transient expression of Cas9 and sgRNAs targeting endogenous marker gene PKS12
Source: Fungal Biol Biotechnol. 2021 Nov 17;8:15. doi: 10.1186/s40694-021-00121-8 (PMC8597179; doi:10.1186/s40694-021-00121-8)
Supplement: Supplementary file 11 — Additional file 11: Table S11. Method for making Potato Sucrose Agar (PSA). [file 40694_2021_121_MOESM11_ESM.docx]

**Additional File 11**

**Table S11: Method for making Potato Sucrose Agar (PSA)**

Ingredients 1 litre

Potato (main crop) 200 g

Sucrose 20 g

Purified agar 20 g

*Method*

- Peel and dice the potatoes (approximately 15-20 mm^3^)
- Boil for 10 mins then filter through gauze
- Add the sucrose to the filtrate, stir to dissolve and make to litre using purified water
- Allow to cool
- Adjust the pH to 6.5 using NaOH
- Aliquot if required
- Add the agar and stir/shake to suspend
- Autoclave to sterilise

(Personal communication, Marlow Foods)
